# Identification and Assessment of Necroptosis-Related Genes in Clinical Prognosis and Immune Cells in Diffuse Large B-Cell Lymphoma

**DOI:** 10.3389/fonc.2022.904614

**Published:** 2022-06-22

**Authors:** Qikai Zhang, Zongsi Zhu, Jiaqiang Guan, Cuiping Zheng

**Affiliations:** ^1^ Department of Hematological Oncology, Wenzhou Central Hospital, The Dingli Clinical Institute of Wenzhou Medical University, Wenzhou, China; ^2^ First Clinical Medical College, Wenzhou Medical University, Wenzhou, China

**Keywords:** necroptosis, diffuse large B-cell lymphoma, prognosis, tumor microenvironment, biomarker

## Abstract

**Background:**

With the unveiling of new mechanisms and the advent of new drugs, the prognosis of diffuse large B-cell lymphoma (DLBCL) becomes promising, but some patients still progress to the relapse or refractory stage. Necroptosis, as a relatively novel programmed cell death, is involved in the development of multiple tumors. There are no relevant studies on the prognostic significance of necroptosis in DLBCL to date.

**Methods:**

We identified the differential necroptosis-related genes (NRGs) by comparing the DLBCL and normal control in GSE12195 and GSE56315 datasets. TCGA DLBC and GSE10846 containing clinical information and microarray expression profiling were merged as the entire cohort. We performed consensus clusters based on NRGs and two clusters were obtained. Kaplan–Meier (K-M) survival analysis, GSVA, GO, KEGG, and ssGSEA were used to analyze the survival, function, and immune microenvironment between two clusters. With LASSO and proportional hazard model construction, we identified differentially expressed genes (DEGs) between NRG clusters, calculated the risk score, established a prognostic model, and validated its value by calibration and ROC curves. The entire cohort was divided into the training and test cohort, and GSE87371 was included as an external validation cohort. K-M, copy number variation, tumor mutation burden, and drug sensitivity were also analyzed.

**Results:**

We found significant differences in prognosis between the two NRG clusters. Cluster A with a poor prognosis had a decreased expression of NRGs and a relatively suppressed immune microenvironment. GSVA analysis indicated that cluster A was related to the downregulation of the TGF-β signaling pathway and the activation of the Notch signaling pathway. The risk score had an accurate predictive ability. The nomogram could help predict the survival probability of DLBCL patients in the entire cohort and the external validation cohort. The area under the curve (AUC) of the nomogram, risk score, and International Prognostic Index was 0.723, 0.712, and 0.537, respectively. γ/δ T cells and Macrophage 1 cells decreased while Macrophage 2 cells and Natural Killer resting cells increased in the high-risk group. In addition, the high-risk group was more sensitive to the PI3K inhibitor and the PDK inhibitor.

**Conclusion:**

We explored the potential role of necroptosis in DLBCL from multiple perspectives and provided a prognostic nomogram for the survival prediction of DLBCL. Necroptosis was downregulated and was correlated with an immunosuppressed tumor microenvironment and poor prognosis in DLBCL. Our study may deepen the understanding and facilitate the development of new therapy targets for DLBCL.

## Introduction

The important role of apoptosis in the initiation and development of tumors has been well documented in previous studies (1–4). However, when apoptotic resistance occurs, necroptosis could be a possible alternative. Necroptosis is a type of programmed cell death that is similar to necrosis and apoptosis. It is mainly induced by tumor necrosis factor α (TNFα) and the CD95 receptor/Fas ligand complex and recruited *via* TNFR1, leading to various binding forms of receptor-interacting serine/threonine-protein kinase 1 (RIPK1), RIPK3, and mixed lineage kinase ligand (MLKL) and causing cell death eventually (5, 6). Among them, RIPK3 and MLKL matter most. Necroptosis participates in the human development and maintenance of homeostasis as well as tumorigenesis. However, the role of necroptosis in tumors is very complicated, and some viewpoints believe that the cell death caused by necroptosis triggers adaptive immunity (7). However, the relationship of necroptosis itself with tumors or with its immune regulation is not clear so far. On the one hand, necroptosis key regulators are usually downregulated in some cancer cells, suggesting that tumors can escape from death by inhibiting the necroptosis pathway. For example, RIPK3 was significantly decreased in breast cancer (8, 9), colorectal cancer (10), acute myeloid leukemia (AML) (11), and melanoma (12). Increased driver gene mutations in AML mice after RIPK3 knockout promoted leukemogenesis and poor prognosis (11). These studies suggest that RIPK3 may play an antitumor role, but in some other tumors, the expression of necroptosis-related genes (NRGs) is elevated to inhibit their growth. In ovarian cancer treated by cisplatin, disease-free survival time is prolonged in the high MLKL expression group (13). Pancreatic cancer patients with higher expression of MLKL had higher overall survival (OS) and progression-free survival rates (14). Shikonin inhibited lung metastases by inducing RIPK1- and RIPK3-dependent necroptosis in osteosarcoma (15), or regulated the production of reactive oxygen species (ROS) through RIPK3 to inhibit tumor cell proliferation and metastasis (16). Furthermore, RIPK3 promoted TNF-induced ROS by activating multiple enzymes and the ROS bursts inhibited metastasis by killing cancer cells (17). It is also nonnegligible that excessive necroptosis-related factors can, in turn, promote tumorigenesis and metastasis. RIPK1 was upregulated in glioblastoma, which related to poor prognosis (18). In pancreatic cancer, RIPK1, RIPK3, FADD, and MLKL were all increased, accompanied by accelerated cancer cell growth (19).

Diffuse large B-cell lymphoma (DLBCL) is the most common type of non-Hodgkin’s lymphoma (NHL). It can be subdivided into several types based on its origin or genes. The RCHOP regimen has been a classic therapy for DLBCL and has prolonged the survival time to a great extent. The emergence of many new treatments like chimeric antigen receptor T-cell therapy also benefits the patient. Nonetheless, DLBCL remains incurable, and thus, more efforts are needed to confront the challenge.

The current understanding of the relationship between necroptosis and lymphoma is still under exploration. MLKL mRNA inhibits lymphoma growth in mice with human adaptive immunity (20). Locatelli et al. found that the combination of Givinostat (a histone deacetylase inhibitor) and Sorafenib (an RAF/MEK/ERK inhibitor) promoted the production of ROS continuously and activated necroptotic cell death in Hodgkin’s lymphoma (HL) (21). Casagrande et al. also suggested that Trabectedin promoted the necroptosis of HL (22). In NHL, a single-nucleotide polymorphism (SNP) of RIPK3 has been uncovered (23). Immunotoxin containing saporin-S6 was cytotoxic, which could be partially prevented by necrostatin-1 (a necroptosis inhibitor) (24). The function of bortezomib is clear in myeloma. Bhatti et al. reported that bortezomib combined with Smac mimetics BV6 could increase the phosphorylation of MLKL and induce NHL cell death (25). Koch et al. found that after the knockout of MLKL in Burkitt’s lymphoma (BL), necroptosis induced by zVAD-fmk was forbidden. Moreover, the activity of MLKL was associated with promoter methylation (26). These studies indicate that necroptosis played a role in lymphoma, but the association between necroptosis and DLBCL is scarce. There was only one article that mentioned that Thymoquinone promoted the development of necroptosis in DLBCL and was more selective than chemotherapeutic agents (27).

In summary, the current understanding of the role of necroptosis in DLBCL remains very superficial, and there is no literature that reveals the relationship between NRGs and DLBCL prognosis. Therefore, we collected all the current genes related to necroptosis and the data from public databases, and used a bioinformatics approach to investigate the underlying function of necroptosis in DLBCL. We developed a prediction model based on these genes and clinical characteristics and tried to clarify the relationship between necroptosis and the immune tumor microenvironment (TME) surrounding DLBCL. Our study initially explored the potential mechanisms of NRGs in DLBCL, showed drug sensitivity for patients in different risk groups, and provided clinicians with a more accurate survival prediction model.

## Materials and Methods

### Data Downloading and Screening

We retrieved the raw transcriptome profiling and clinical data of DLBCL patients and cell lines from The Cancer Genome Atlas (TCGA; https://portal.gdc.cancer.gov/) and the Gene Expression Omnibus (GEO; http://www.ncbi.nlm.nih.gov/geo). The data without survival time were removed from the data. Expression profiling by microarray of DLBCL and normal B-cell lines was obtained from the GSE12195 (73 DLBCL samples versus 20 normal tonsil samples) and GSE56315 (55 DLBCL samples versus 33 normal tonsil samples) datasets with Affymetrix Human Genome U133 Plus 2.0 Platform. Forty-eight DLBCL samples from TCGA DLBC and 305 from GSE10846 (with Affymetrix Human Genome U133 Plus 2.0 Platform) with complete transcriptional and clinical data were preserved for later analysis. Then, we merged the transcriptional expression of TCGA DLBC and GSE10846 into one dataset and identified it as the entire cohort after the removal of the batch effect using the “limma” package (28). Batch effects were corrected by the ComBat algorithm in the sva package (29). The entire cohort was divided into the training and test cohorts equally by the “caret” package (30). Furthermore, GSE87371 including 223 patients with Affymetrix Human Genome U133 Plus 2.0 Platform was attained as an external validation cohort. We also downloaded simple nucleotide variant (SNV) data of DLBCL from TCGA DLBC, and copy number variation (CNV) data from UCSC Xena (https://xenabrowser.net/datapages/).

### Cluster Analysis of the Entire Cohort Based on the Necroptosis-Related Genes

We searched “necroptosis” in PubMed (https://pubmed.ncbi.nlm.nih.gov/) and filtered out 198 NRGs from several articles (5, 6, 31, 32). All NRGs are listed in **Supplementary Table 1**. With the help of the “limma” and “reshape2” packages (33), we compared the expression of DLBCL and normal cell lines and identified 118 NRGs in GSE12195 and GSE56315. Among the 118 NRGs, 103 NRGs were identified as the shared genes in the entire cohort. Then, we performed Cox analysis and found 25 NRGs that were correlated with survival (*p* < 0.05) using the “limma”, “survival” (34), and “survminer” packages (35). To explore their connections, the gene network and protein–protein interaction (PPI) were generated by the “igraph” package (36) and STRING (https://cn.string-db.org/). Based on the 25 NRGs, two clusters (named NRG clusters) were defined by the “ConsensusClusterPlus” package. We also drew the Kaplan–Meier (K-M) survival analysis curve using the “survival” and “survminer” packages. Principal component analysis (PCA) plotted by the “limma” and “ggplot2” packages (35) could assess the precision of NRGs. Heatmap including clusters and various clinical data was drawn using the “pheatmap” package (37). Furthermore, we ran the “GSVA” (38) and “GSEABase” packages (39) to clarify the relationship of function and immune cells with 25 NRGs.

### Identification and Assessment of the Differentially Expressed Genes in NRG Clusters

The univariate analysis of differentially expressed genes (DEGs) associated with prognosis in NRG clusters was performed using the “limma” package. For these DEGs, we conducted Gene Ontology (GO) and Kyoto Encyclopedia of Genes and Genomes (KEGG) analysis using the “clusterProfiler” package (40). Later, to make it more accurate, we obtained 24 DEGs after the Least Absolute Shrinkage and Selection Operator (LASSO) regression and Cox proportional hazard model construction by the “survival”, “survminer”, and “timeROC” packages (41). The risk score of each sample was calculated based on the expression of the DEGs. The risk score = Coef1*Exp1 + Coef2*Exp2 +…+ Coef24*Exp24. The entire cohort was subdivided into low- and high-risk groups by the median value of risk score. We then drew the K-M curve, risk curve, and survival status curve of the entire, training, test, and external validation cohort by “pheatmap”. In order to estimate its predictive value, we drew the receiver operating characteristic (ROC) curve at 1, 3, and 5 years using the “survival”, “survminer”, and “timeROC” packages. Meanwhile, cluster analysis was performed again based on the DEGs using the “ConsensusClusterPlus” package (42) and the K-M curve of two clusters (named DEG clusters) was made using the “survival” and “survminer” packages. The Sankey diagram between NRG clusters, risk score, DEG clusters, and clinical information was drawn using “limma”, “ggpubr” (https://CRAN.R-project.org/package=ggpubr), and “ggalluvial” (https://CRAN.R-project.org/package=ggalluvial).

### Construction and Evaluation of Nomogram

To improve the accuracy of the current prognosis score system, we performed univariate and multivariate Cox regression analysis of risk score and clinicopathological characteristics using the “survival” package. The factors with *p*-value < 0.05 were contained to draw the nomogram, calibration curve, and ROC curve using the “survival” package. As there were two regimens in the dataset, we divided them into the CHOP and RCHOP groups and drew the ROC curves. To evaluate the stability of the predictors in the nomogram, we perform collinearity diagnosis by SPSS.

### Tumor Mutation Burden, CNV, Immune Infiltration, and Drug Sensitivity Analysis Between Low- and High-Risk Groups in the Entire Cohort

We performed the “maftools” package (43) to show the TMB and used “Rcircos” (44) to identify the CNV and its location on the chromosome for all NRGs in TCGA DLBC. To determine the immune cells related to DEGs and risk score, CIBERSORT algorithm was employed. The TMB was mapped and evaluated by the “maftools” package between the low- and high-risk groups, so as the TMB and risk score. To figure out whether the risk score affected drug selection, we used the “pRRophetic” package (45) to pick out the targeted drugs between the low- and high-risk groups.

### Statistical Analysis

All analyses were conducted by using the R software (version 3.4.0) and SPSS software (version 24.0). The references of all packages were all cited. K-M and Cox regression were used to identify the prognosis-related NRGs. We compared statistical differences between the two groups by the Wilcoxon test. We performed survival analysis by the K-M method and analyzed the difference of OS by log-rank test. LASSO and the Cox proportional hazard model were used to identify the most significant genes. We performed univariate and multivariate analysis and built the nomogram by the Cox regression method. The sensitivity and specificity of the nomogram were implemented by the calibration curve and the area under the curve (AUC) of the ROC curve. Collinearity analysis was achieved by the collinearity diagnosis. Statistical significance was defined as *p* < 0.05 (unless otherwise specified).

## Results

### The Genetic Mutation Landscape and the CNV Location of NRGs in DLBCL

**Figure 1:**
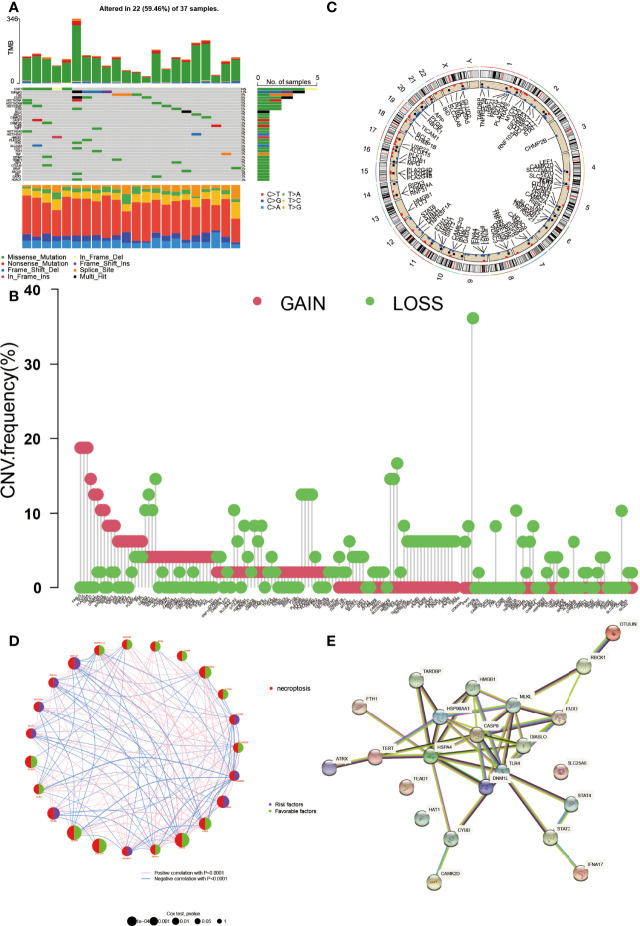
The genetic landscape of NRGs in DLBCL. **(A)** The tumor mutation burden frequency and classification of NRGs in DLBCL. **(B)** The copy number variation (CNV) frequency of NRGs in DLBCL. The height represents the alteration frequency. **(C)** The location of the CNV alteration on chromosomes. **(D)** The gene interaction network of NRGs in DLBCL. The circle size indicates its significance. The right part of the circle represents its role, purple for risk factor and green for favorable. The line between genes means they were correlated with each other (*p* < 0.0001). **(E)** The protein–protein interaction encoded by NRGs in DLBCL by STRING.

The clinical characteristics of the GEO and TCGA datasets are summarized in **Table 1**. The expression, genetic mutation landscape, and the CNV location of NRGs in DLBCL are shown in **Figure 1**. There were 118 NRGs that were significantly differentially expressed between normal cells and DLBCL (*p* < 0.05) (see **Supplementary Figure 1**). Among the 118 NRGs, 34 of them were mutated in 59.46% (22/37) of DLBCL samples (**Figure 1A**). STAT3 was the most frequent mutated NRGs (14%) followed by TNFAIP3, FAS, and STAT6 (11%, 8%, and 8%, respectively). It was not hard to find that the missense mutation was the most common mutation variant, and C>T was the highest SNV class. As for the CNV, nearly all of them were revealed as gain or loss after CNV analysis (**Figure 1B**). CDKN2A, TNFAIP3, BACH2, and MAP3K7 were the most deleted NRGs while FASLG, GLUL, PLA2G4A, and USP21 gained the most. Then, we mapped the exact location of the CNV alteration on chromosomes (**Figure 1C**), which mainly enriched in chromosomes 1, 2, and 9. We identified 25 NRGs according to the gene expression, survival time, and follow-up status by Cox analysis. The K-M curve based on 25 genes is shown in **Supplementary Figure 2**. Moreover, the gene network is shown in **Figure 1D**. The size of each circle indicated its significance in DLBCL. The right part of the circle represented its role in DLBCL. Purple meant it was a risk factor while green meant it was favorable. The line between genes indicated that they were correlated with each other (*p* < 0.0001). Meanwhile, we also explored the relationship between the proteins encoded by the NRGs (**Figure 1E**). CASP8 could interact with other proteins greatly.

**Table 1:** The clinical characteristics of the GEO and TCGA datasets.

Characteristics	Training cohort (GSE10846)	TCGA DLBC	Validation cohort (GSE87371)
Sample size	305	48	221
Age, years mean (SD)	60.43 (15.21)	56.27 (13.80)	56.71 (16.08)
Gender			
Female	134	26	105
Male	171	22	116
Stage			
1	50	8	29
2	94	17	42
3	68	5	35
4	93	12	115
unknow	–	6	–
≥2 Extranodal sites	23	–	–
ECOG PS>1	75	–	–
LDH>ULN	146	–	–
Cell of origin			
GCB	133	–	84
Non-GCB	172	–	137
Overall survival			
Time, years mean (SD)	3.36 (3.04)	3.69 (3.98)	2.92 (1.47)
Death	122	9	53

TCGA, The Cancer Genome Atlas; ECOG PS, The Eastern Cooperative Oncology Group performance score; ULN, upper limit of normal; GCB, germinal center B-cell-like subtype.

### Establishment and Analysis of the NRG Clusters

**Figure 2:**
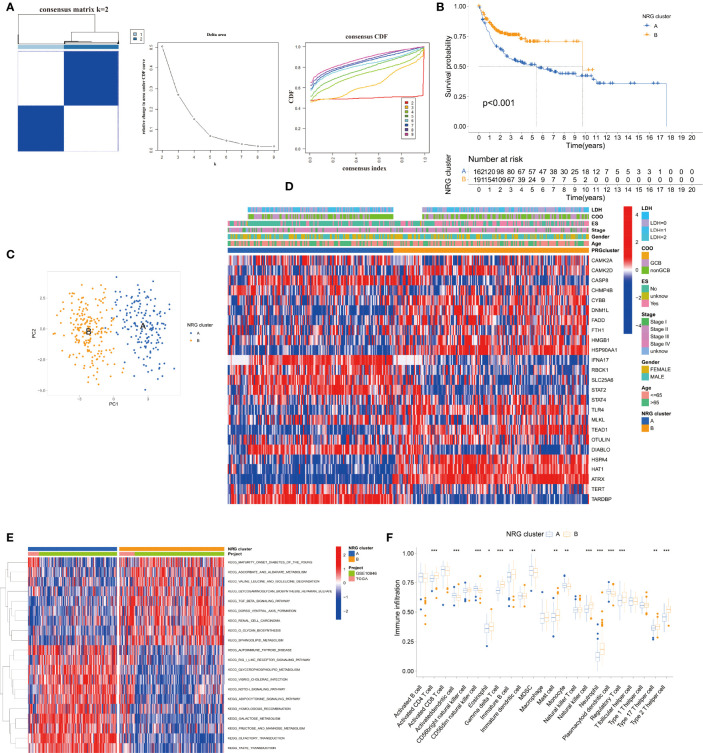
Establishment and analysis of the NRG clusters. **(A)** The consensus matrix, delta area, and consensus CDF by cluster analysis based on NRGs. Two clusters would be best. **(B)** Kaplan–Meier survival curves of two NRG clusters (*p* < 0.001). **(C)** Principal component analysis of NRG clusters. **(D)** Heatmap of NRG clusters and clinical data of DLBCL patients. Data of lactate dehydrogenases (LDH) and cell of origin (COO) are missing. **(E)** KEGG pathway enrichment analysis between NRG clusters by GSVA. **(F)** Immune infiltration between NRG clusters by ssGSEA. *p < 0.05, **p < 0.01, ***p < 0.001.

According to the 25 NRGs, we established the NRG clusters from the TCGA DLBC and GEO datasets using the “ConsensusClusterPlus” package (**Figure 2A**). To evaluate the prognosis value of the cluster, we drew the K-M curve, PCA, and heatmap (**Figures 2B–D**). **Figure 2B** shows that cluster A had a worse outcome than B (*p* < 0.001). PCA could easily distinguish cluster A from cluster B. The link between gene expression, NRG clusters, and clinical characteristics is shown in **Figure 2D**. Moreover, we exploited the KEGG pathway and immune cells based on the 25 NRGs by gene set variation analysis (GSVA) and single-sample Gene Set Enrichment Analysis (ssGSEA) as well (**Figures 2E,F**). In cluster A, the downregulated genes were enriched in the TGF-β signal pathway and metabolism of glucose, amino acid, and lipid while the overexpressed genes were enriched in the Notch signal pathway, metabolism, and transductions (**Figure 2E**). As for the immune cells in cluster A, activated dendritic cells (DCs), CD56dim natural killer cells (NK cells), myeloid-derived suppressor cells (MDSCs), monocytes, and plasmacytoid DCs were upregulated while activated CD4^+^T cells, γ/δ T cells, immature B cells, mast cells, NK cells, neutrophils, regulatory T cells, type 17 helper T cells, and type 2 helper T cells were downregulated (**Figure 2F**).

### Identification and Assessment of the DEGs Between the NRG Clusters

**Figure 3:**
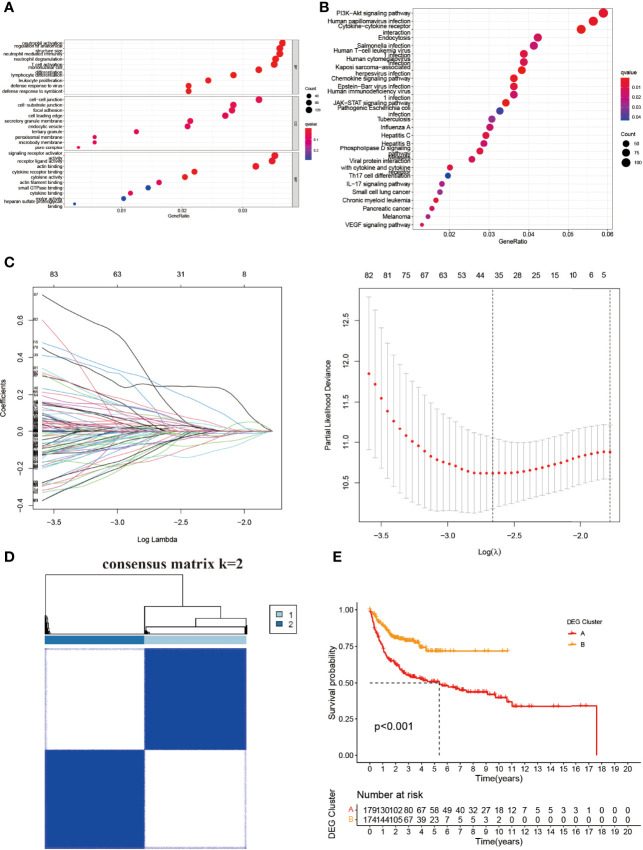
Identification and construction of NRG-based DEG clusters. **(A)** GO and **(B)** KEGG analysis of the differential genes between two NRG clusters. **(C)** LASSO and Cox proportional hazard model construction between NRG clusters. **(D)** The consensus matrix by cluster analysis based on DEGs. Two clusters would be best. **(E)** Kaplan–Meier survival curves of two DEG clusters (*p* < 0.001). BP, biological process; CC, cellular component; MF, molecular function.

To understand the potential significance between the two clusters based on NRGs, we then used the “limma” and “clusterProfiler” packages to clarify the underlying function of DEGs. GO pathway analysis showed that the DEGs were enriched in neutrophil activation, neutrophil-mediated immunity, neutrophil degranulation, T-cell activation, mononuclear cell differentiation, and lymphocyte differentiation in biological process (BP) (**Figure 3A**). The DEGs were mainly involved in the PI3K-Akt signal pathway, cytokine–cytokine receptor interaction, endocytosis, the chemokine signal pathway, the JAK-STAT3 signal pathway, and the NOD-like receptor signal pathway in KEGG analysis (**Figure 3B**). There were 24 DEGs between the NRG cluster (**Figure 3C** and **Supplementary Table 2**). We divided the entire cohort into two DEG clusters (**Figure 3D** and **Supplementary Figure 3**). K-M showed a significant difference between the DEG clusters (C, D) (*p* < 0.001) (**Figure 3E**), indicating the potential of DEGs in DLBCL.

**Figure 4:**
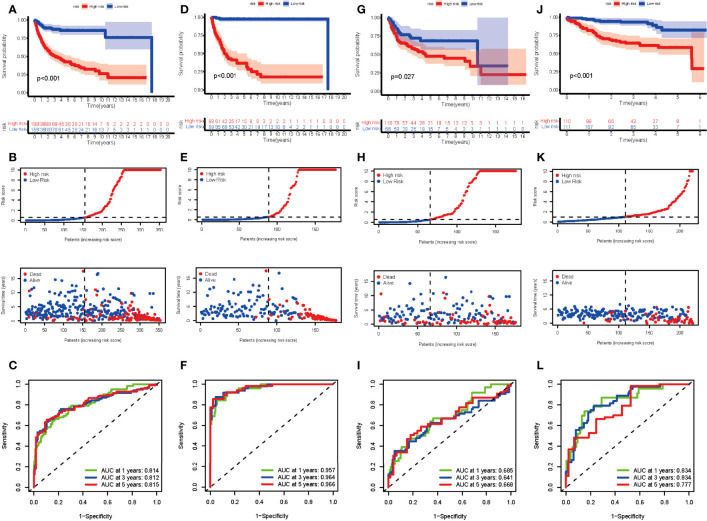
Evaluation of the risk score in the entire, training, test, and external validation cohort. **(A**, **D, G**, **J)** K-M curve of the entire, training, test, and external validation cohort, respectively. The *p*-value is <0.001, <0.001, 0.027, and <0.001, respectively. **(B**, **E**, **H**, **K)** The risk curve and survival status curve of each cohort, respectively. **(C**, **F**, **I**, **L)** ROC with AUC at 1, 3, and 5 years of each cohort, respectively.

The entire cohort was randomly assigned into the training cohort and test cohort to examine the predictive potential of DEGs. After calculating the risk score, we divided the entire cohort and GSE87371 into the low- and high-risk groups. The K-M curve suggested a significant difference in the entire cohort, training cohort, and test cohort (**Figures 4A,D,G**). The risk curve and survival status curve showed that it could separate the high-risk group from the other (**Figures 4B,E,H**). To assess the prognostic value further, we plotted the ROC curve. The AUCs were 0.814, 0.812, and 0.815 at 1, 3, and 5 years, respectively, in the entire cohort (**Figure 4C**). For the training cohort, the AUCs were 0.957, 0.964, and 0.977, respectively (**Figure 4F**). AUCs in the test cohort were 0.685, 0.641, and 0.668 at 1, 3, and 5 years, respectively (**Figure 4I**). In the external validation cohort, the risk score could distinguish between the low- and high-risk groups (*p* < 0.001) (**Figures 4J,K**) with AUCs of 0.834, 0.834, and 0.777 at 1, 3, and 5 years, respectively (**Figure 4L**). These results elucidated that the DEG-based risk score could perfectly predict the clinical outcome of DLBCL patients.

### Construction and Evaluation of the DEG-Related Prognostic Signature

**Figure 5:**
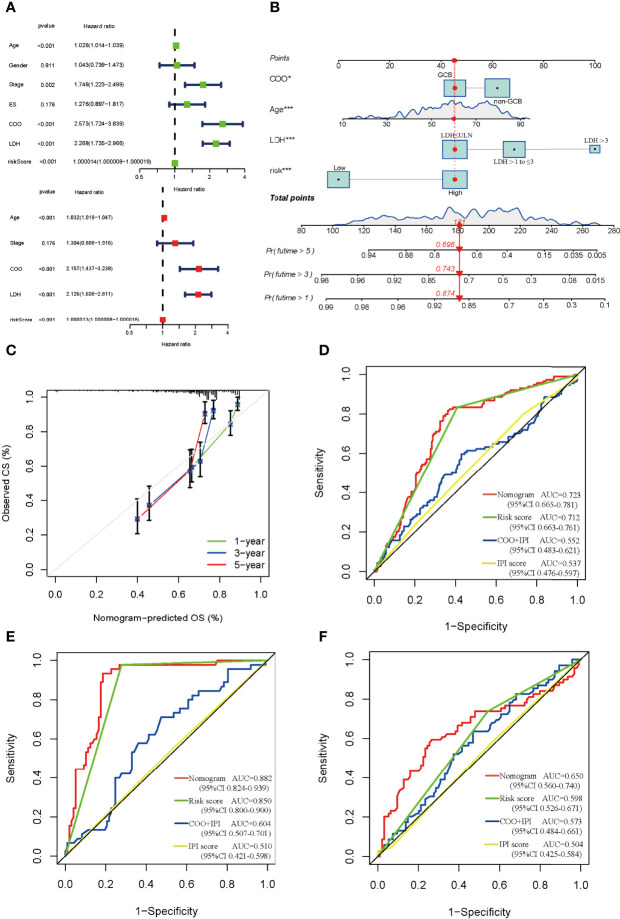
Construction and estimate of nomogram. **(A)** The univariate (green) and multivariate (red) Cox regression analysis of clinical characteristics and risk score. **(B)** The nomogram integrating COO, age, LDH, and risk score for DLBCL patients. **(C)** The calibration curve of the nomogram at 1, 3, and 5 years, respectively. **(D)** ROC curve of the nomogram, risk score, COO+IPI, and IPI score. ROC curves of the nomogram, risk score, COO+IPI, and IPI score in the CHOP regimen group **(E)** and RCHOP regimen group **(F)**. ES, extranodal sites; COO, cell of origin; LDH, lactate dehydrogenases; GCB, germinal center B-cell like; ULN, upper limit of normal.

The univariate and multivariate Cox regression analysis indicated that age, cell of origin (COO), LDH, and risk score served as the independent prognostic factors (**Figure 5A**), and we constructed the nomogram based on them (**Figure 5B**). The nomogram predicted that OS at 1, 3, and 5 years was approximately close to the observed OS in the calibration curve (**Figure 5C**). To assess the factors in the nomogram, we performed collinearity diagnosis and the results showed that the collinearity tolerance of each predictor was great than 0.1 and the variance inflation factor was less than 10. Condition index was less than fifteen, and the maximum value in the horizontal row was not simultaneously greater than 0.5 in the vertical column (**Supplementary Tables 3**, **4**). These results indicated that there was no collinearity in the independent predictors. Compared with the COO plus International Prognostic Index (IPI, scoring with available data) or IPI alone, the nomogram could remarkably increase the AUC (the AUC is 0.723, 0.712, 0.552, and 0.537, respectively), suggesting its vital role in the prognosis of DLBCL (**Figure 5D**). Different regimens for DLBCL have an effect on the prognosis as well. Our results showed that the risk score could markedly enhance the efficacy in both CHOP and RCHOP groups (**Figures 5E,F**).

### Immune Cells, CNV, TMB, and Drug Sensitivity of the NRGs Between Low- and High-Risk Groups

**Figure 6:**
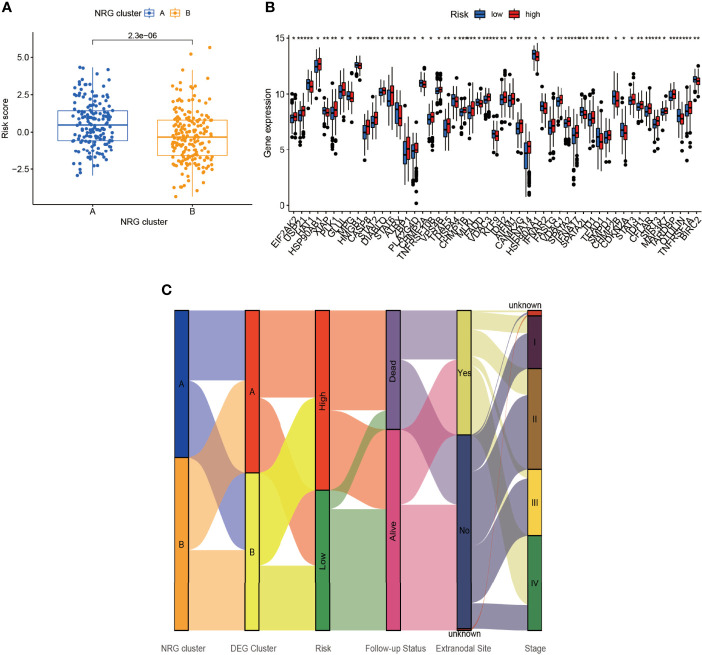
Connection of risk score and NRG clusters. **(A)** The relationship of risk score and NRG clusters (*p* = 2.3×10^-6^). **(B)** The differential NRGs between the low- and high-risk group. **(C)** Sankey diagram including NRG clusters, DEG clusters, risk score, follow-up status, extranodal site, and stage. *p < 0.05, **p < 0.01, ***p < 0.001.

By comparing the risk score between NRG clusters, we found that the risk score of cluster A was significantly higher than that of cluster B (*p* = 2.3e-06) (**Figure 6A**). As seen in **Figure 6B**, several NRGs showed a significant difference between the low- and high-risk group. Apart from that, we plotted the Sankey diagram of NRG clusters, DEG clusters, risk score, follow-up status, extranodal site, and Ann Arbor stage (**Figure 6C**). The NRG clusters, DEG clusters, risk score, and follow-up status corresponded well while the clinical characteristics varied widely.

**Figure 7:**
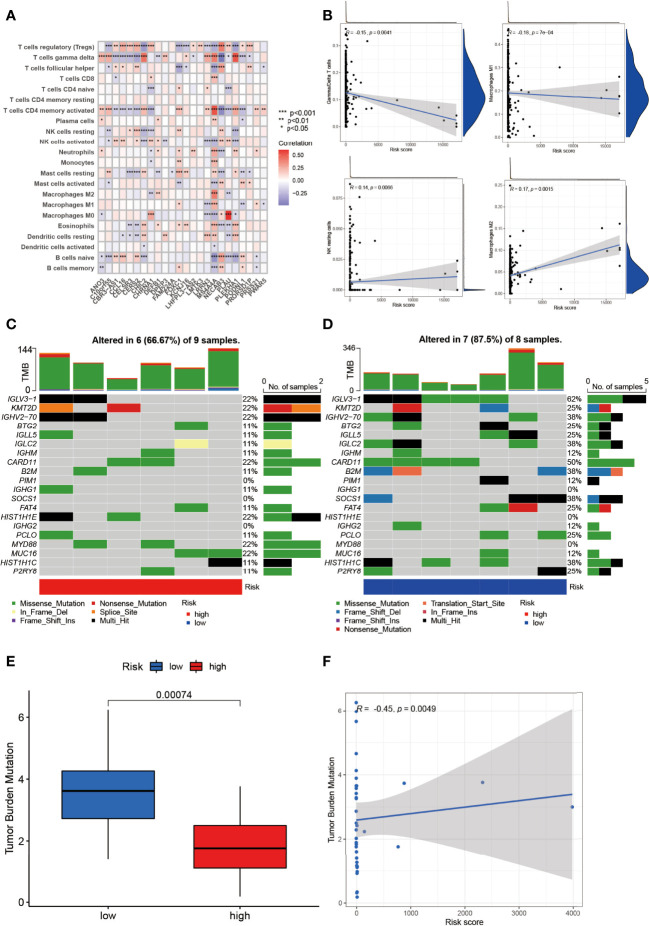
Identification of immune infiltration and genetic variants underlying the different risk groups. **(A)** The correlation intensity between DEGs and immune cells. **(B)** The relationship of immune cells in risk score. γ/δ T cell and Macrophage 1 were negatively correlated with risk score while NK resting cell and Macrophage 2 were positively correlated (*p* < 0.01). The CNV frequency of **(C)** high-risk and **(D)** low-risk groups. **(E)** The TMB between the low- and high-risk group (*p* = 0.00074). **(F)** The correlation of TMB and risk score (*p* = 0.0049). *p < 0.05, **p < 0.01, ***p < 0.001.

The relationship between NRGs and immune cells had been demonstrated before, and we herein investigated the correlation between DEGs and immune cells. In **Figure 7A**, T regulatory cells (Treg), γ/δ T cells, and CD4 activated memory T cells ranked most with the DEGs, correlated with 18/24 (75%), 18/24 (75%), and 17/24 (70.83%) genes, respectively (**Figure 7A**). Between the low- and high-risk group, γ/δ T cells and Macrophage 1 (M1) were negatively correlated with risk score while NK resting cells and Macrophage 2 were positively correlated (**Figure 7B**).

In addition, the CNV of NRGs in the whole samples had been displayed above, but they varied between the low- and high-risk group. As was shown in **Figure 7C**, IGLV3-1, KMT2D, GHV2-70, CARD11, HIST1H1E, MYD88, and MCU16 mutated the most (22%) with 6/9 (66.67%) altered samples in the high-risk group. Intriguingly, IGLV3-1 had the highest mutation (62%), followed by CARD11 (50%), GHV2-70 (38%), IGLC2 (38%), B2M (38%), SOCS1 (38%), and HIST1H1C (38%) with 7/8 (87.5%) altered samples in the low-risk group (**Figure 7D**). Missense mutation was the most common variant classification in both groups.

TMB was a pivotal part in tumors as well. NRG-related TMB appeared to be different apparently between the low- and high-risk groups (**Figure 7E**). In DEG clusters, NRG-related TMB was positively related to risk score (**Figure 7F**).

**Figure 8:**
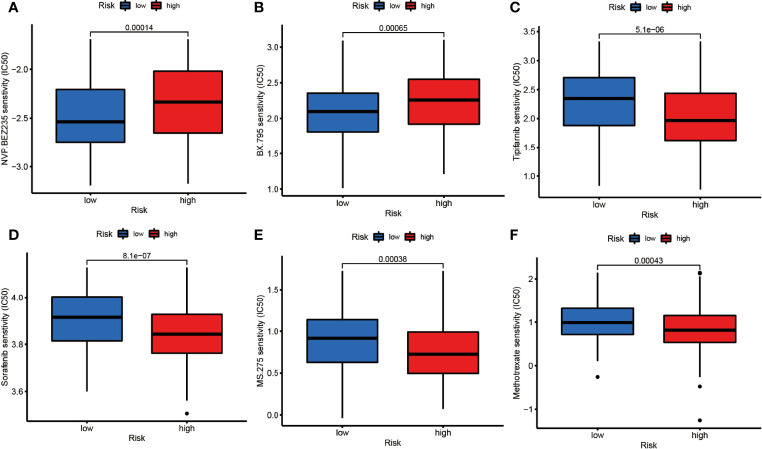
The drug sensitivity of **(A)** NVP.BEZ235, **(B)** BX795, **(C)** Tipifarnib, **(D)** Sorafenib, **(E)** MS.275, **(F)** Methotrexate between the low- and high-risk groups (p<0.01).

To testify the therapeutic potential of DEGs, we performed the correlation of different risk groups with current anti-tumor drugs. Twenty drugs were discovered between the low- and high-risk group (**Figure 8** and **Supplementary Figure 4**). We found that NVP-BEZ235 and BX.795 were sensitive in the high-risk group (**Figures 8A,B**) while the rest were more sensitive in the low-risk group.

## Discussion

Previously, necroptosis was considered to be an accidental form of programmed death that was not controlled by molecular events. However, necroptosis is similar to apoptosis in that they are largely intertwined in cells and may even overcome apoptotic resistance by immunity (6). Several studies have shown that necroptosis is associated with tumors (6, 46). However, studies on necroptosis in DLBCL are limited. Here, we established a molecular signature based on NRGs that was significantly associated with the survival time of DLBCL in both the entire cohort and the external validation cohort by bioinformatics methods. The nomogram combined with the genes and clinical information could largely improve the efficacy of the prognosis for DLBCL patients. Models that cover both clinical and gene expression data will accurately provide instructions for the classification and therapy of DLBCL patients.

By comparing the expression of NRGs in normal and DLBCL cell lines, many NRGs with differential expression were identified. MLKL, which is very specific to necroptosis, is one of the components for the assembly of the necroptosis complex. Decreased MLKL is associated with shorter OS in many cancers (13, 14, 47–49), which may be related to the immune surveillance of the TME. Since DLBCL is a highly heterogeneous tumor, we divided the entire cohort into two clusters based on differential NRGs. In cluster A, most of the NRGs were downregulated and were associated with a poor prognosis, while in cluster B, the expression was increased and was related with a relatively favorable prognosis. MLKL, which is very specific for necroptosis, is one of the components underlying the assembly of the necroptosis complex. Decreased MLKL was associated with shorter OS in many cancers (13, 14, 47–49), which was consistent with our results. Further analysis showed that MLKL was closely correlated to CASP8 in cluster A. One of the triggers of necroptosis is that if caspase 8 activity is inhibited, complex IIb binds RIPK1 to phosphorylate RIPK3 and forms complex IIc (necroptosome). The necroptosome recruits and phosphorylates MLKL, resulting in altered conformation, causing MLKL translocation to the cell membrane, increasing cell membrane permeability, and leading to cell death finally (5). Hence, we presumed that the interaction between MLKL and CASP8 was inhibited, leading to the development of DLBCL. However, further investigations are required for verification. KEGG pathway analysis showed that the highly expressed genes in cluster A with poor prognosis were mainly enriched in the Notch signaling pathway, while the lowly expressed genes were enriched in the TGF-β signaling pathway. Notch plays a role in promoting and maintaining lymphoid malignancies by regulating relevant signaling pathways (50). The Notch signaling pathway has been reported to be aberrantly activated in DLBCL (51), and it has also been found that Notch can interact with the tumor or stem cell microenvironment *via* stromal cells (52). Our study found that the Notch signaling pathway enrichment was associated with poor prognosis, suggesting that it may promote tumor development by affecting the necroptosis process of DLBCL or stromal cells in the TME. The TGF-β signaling pathway can inhibit tumor cell proliferation (53). In DLBCL, the pathway was previously reported to be recurrently inactivated (54), but in multiple myeloma and myeloproliferative disorders, TGF-β exerted pro-tumor activity by inhibiting host tumor immune surveillance. Disruption of TGF-β signaling pathway could also promote hematologic tumors by regulating the stroma and immune system (55). Our study suggested that TGF-β was a tumor suppressor in DLBCL. However, the specific mechanism remains to be elucidated.

Necroptosis is tightly related to anti-tumor immunity (56). Werthmöller et al. reported that the combination of the zVAD-fmk (a pan-cysteine aspartate protease inhibitor) with other therapies (including radiotherapy, chemotherapy, and thermotherapy) could induce necroptosis in melanoma. Regulatory T cells (Treg) are reduced and dendritic cells (DC) and CD8+ T-cell infiltration are increased in anti-immunity, resulting in significant suppression of tumor growth (57). The relationship between tumors and immune cells is complicated. Our study found that the levels of activated CD4+T cells, eosinophils, γ/δ T cells, immature B cells, mast cells, NK cells, neutrophils, regulatory T cells, type 17 T helper cells, and type 2 T helper cells were reduced, while activated DCs, CD56 dim NK cells, MDSCs, monocytes, and plasmacytoid DCs were increased in cluster A. We suppose that the expression of NRGs is downregulated in DLBCL with poor prognosis, leading to a reduced sensitivity of these cells to TNFα agonism (11). The reduction of anti-tumor immune cells and the increase of inhibitory immune cells may result in a disturbed immune microenvironment and an immunosuppressive state in DLBCL. The immune microenvironment of DLBCL is distinct from melanoma, and with its unique response characteristics and immune microenvironment, necroptosis-related immunomodulatory agents need to be explored.

Meanwhile, the risk groups based on DEGs may provide guidance for drug selectivity. We found that the high-risk group was more sensitive to both NVP.BEZ235 and BX.795. NVP.BEZ235 is a targeted drug that inhibits PI3K/mTOR, and the most enriched KEGG signaling pathway was exactly PI3K-Akt. This drug has been previously studied in DLBCL cell lines (58), but the relationship between NVP.BEZ235 and necroptosis is unclear. BX.795 targeting PDK1 inhibition, with no relevant research in DLBCL, may provide a potential direction for follow-up studies. Sorafenib was found to promote ROS production in HL, thus causing necroptotic death (21). Our study found that the group with relatively abundant NRGs was more sensitive to it, suggesting that there is some connection between them.

Of course, there are some limitations to our study. First of all, the number of genes we obtained was a little bit high, but these genes were well-analyzed and confirmed to be meaningful. The second is that our study was based on public databases and has not been validated by cellular experiments and clinical samples yet. It would certainly be better if further proof could be provided. The aim is to further clarify the characteristics of each cell population in DLBCL by single-cell sequencing.

## Conclusion

In conclusion, we established a nomogram integrated with clinical characteristics and gene signature based on NRGs that prompted the prediction power of DLBCL. The downregulation of NRGs correlated with a immunosuppressed TME and poor prognosis in DLBCL. Our work may provide a basis for more in-depth studies in the future, a reference for drug selection by clinicians, and a reliable way to predict survival for DLBCL patients.

## Data Availability Statement

The original contributions presented in the study are included in the article/**Supplementary Material**. Further inquiries can be directed to the corresponding author.

## Author Contributions

CZ conceived and designed the research. QZ analyzed and interpreted the results of the data and drafted the paper. ZZ performed the bioinformatics analysis and revised the manuscript. JG prepared figures. All authors contributed to the article and approved the submitted version.

## Funding

This work was supported by the Zhejiang Province Chinese Medicine Science and Technology Program Project (2022ZA167) and the Zhejiang Provincial Medical and Health Science and Technology Project (2022487580).

## Conflict of Interest

The authors declare that the research was conducted in the absence of any commercial or financial relationships that could be construed as a potential conflict of interest.

## Publisher’s Note

All claims expressed in this article are solely those of the authors and do not necessarily represent those of their affiliated organizations, or those of the publisher, the editors and the reviewers. Any product that may be evaluated in this article, or claim that may be made by its manufacturer, is not guaranteed or endorsed by the publisher.

## Supplementary Material

Supplementary Figure 1 The differential NRGs between the DLBCL and normal cell lines in GSE12195 and GSE56315.

Supplementary Figure 2 K-M survival analysis curve of 25 NRGs in entire cohort.

Supplementary Figure 3 The delta area, consensus CDF by cluster analysis based on DEGs.

Supplementary Figure 4 The drug sensitivity between the low- and high-risk group (p<0.01).
