# Supplementary figures and images for: Identification and Assessment of Necroptosis-Related Genes in Clinical Prognosis and Immune Cells in Diffuse Large B-Cell Lymphoma
Source: Front Oncol. 2022 Jun 22;12:904614. doi: 10.3389/fonc.2022.904614 (PMC9257018; doi:10.3389/fonc.2022.904614)

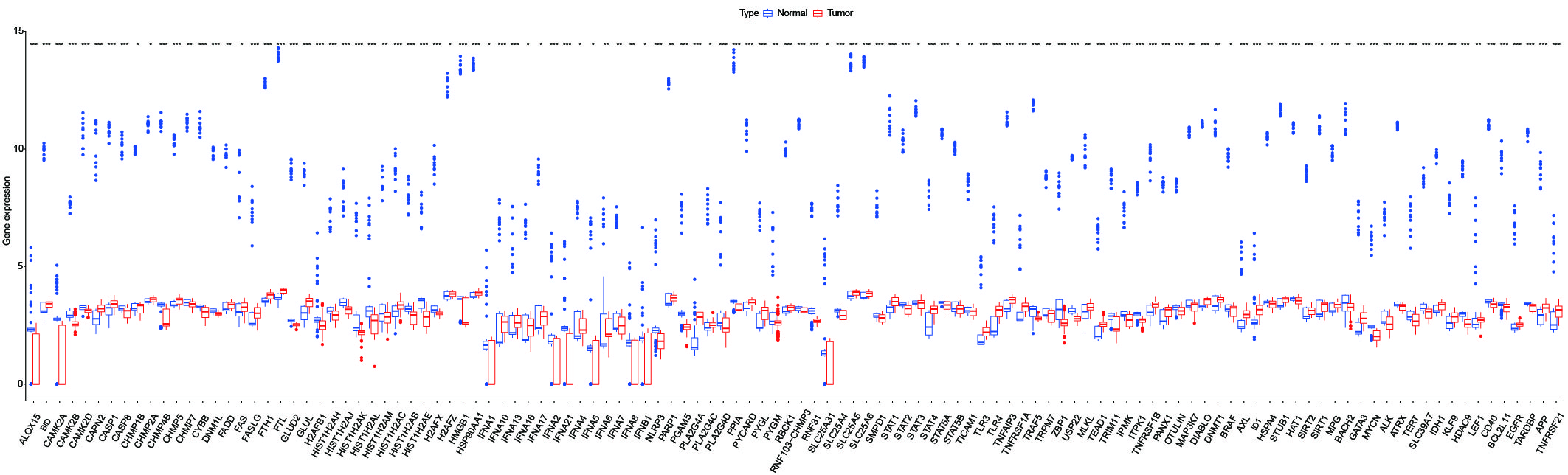

Supplement: Supplementary Figure 1 — The differential NRGs between the DLBCL and normal cell lines in GSE12195 and GSE56315. [file Image_1.jpeg]

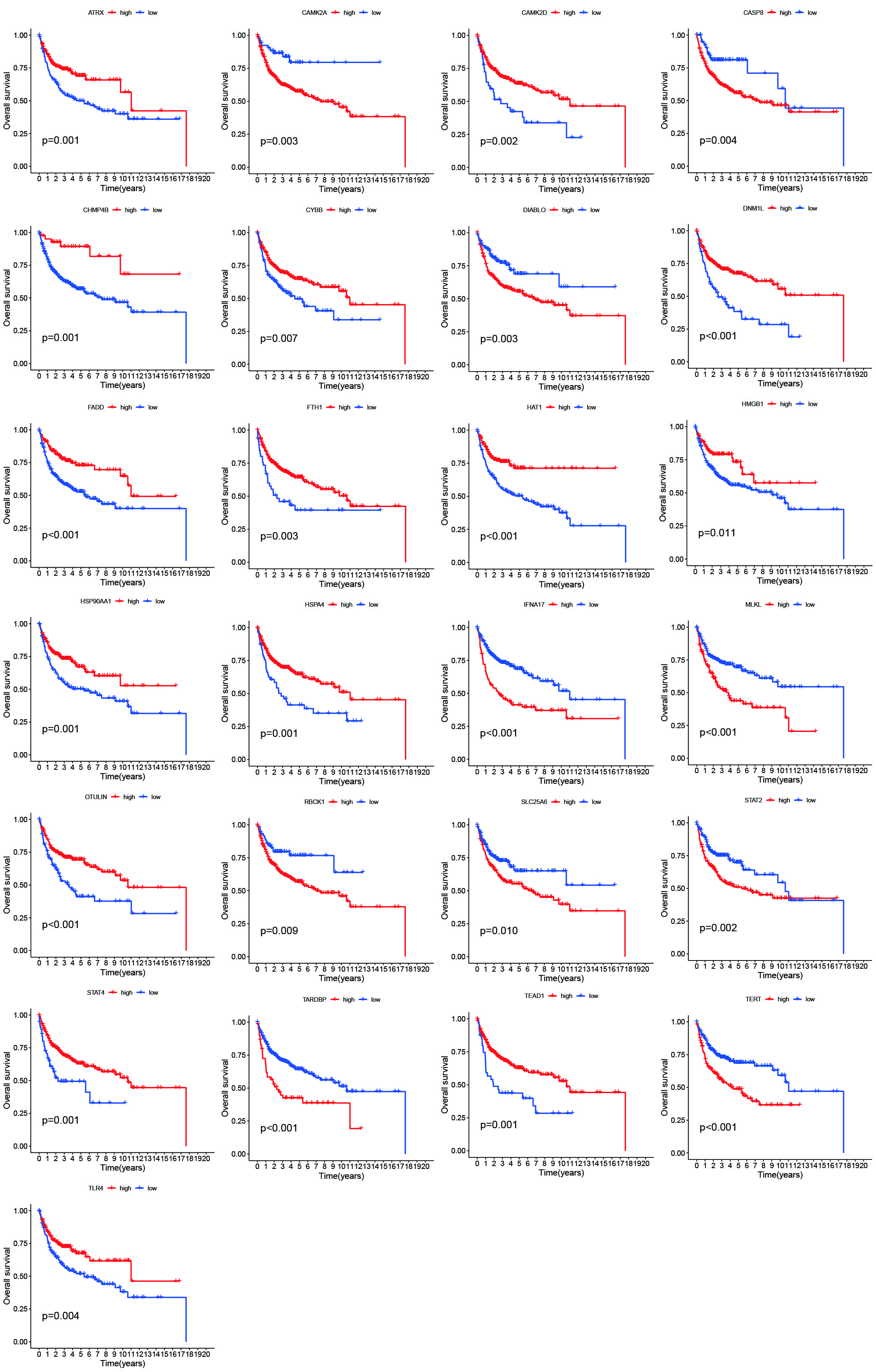

Supplement: Supplementary Figure 2 — K-M survival analysis curve of 25 NRGs in entire cohort. [file Image_2.jpeg]

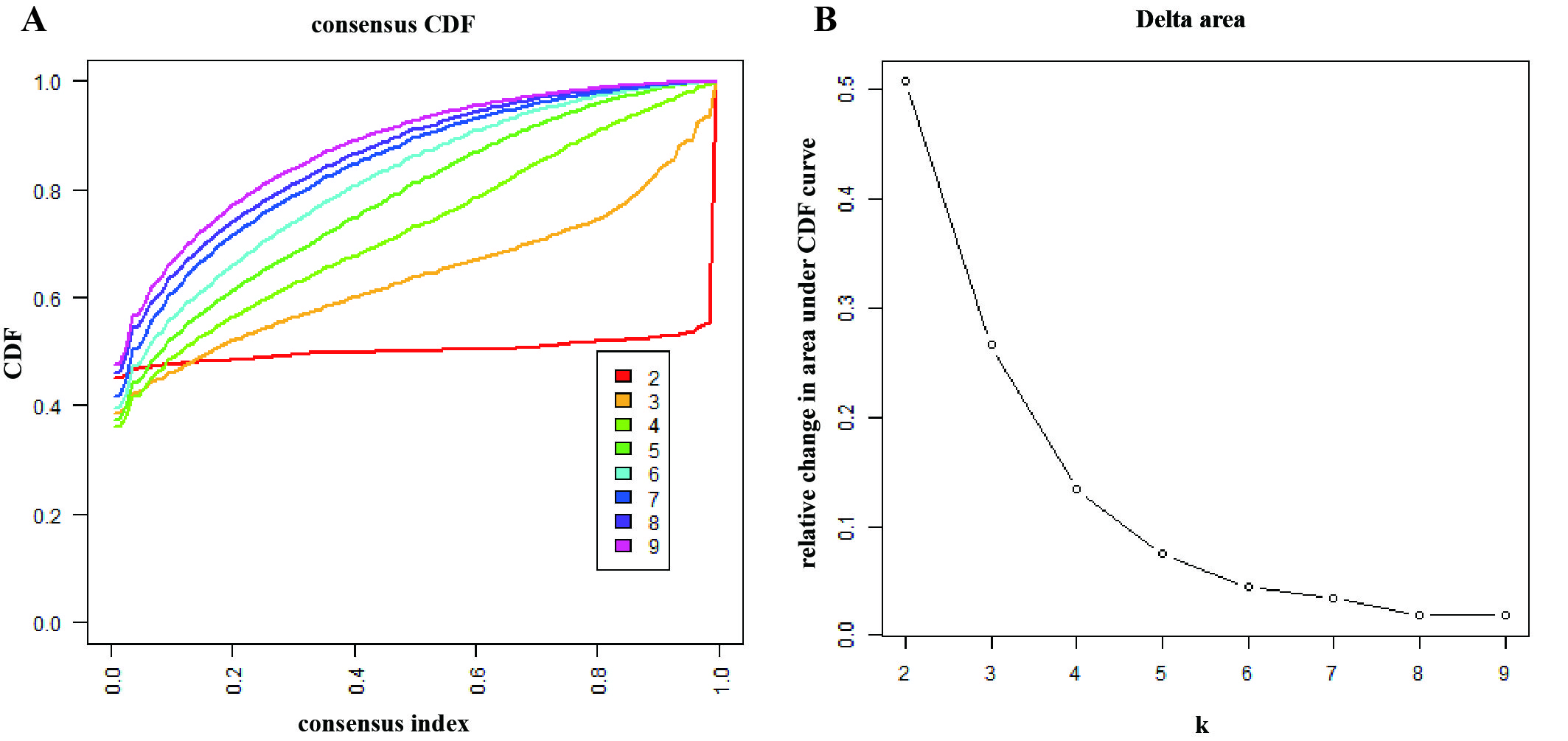

Supplement: Supplementary Figure 3 — The delta area, consensus CDF by cluster analysis based on DEGs. [file Image_3.jpeg]

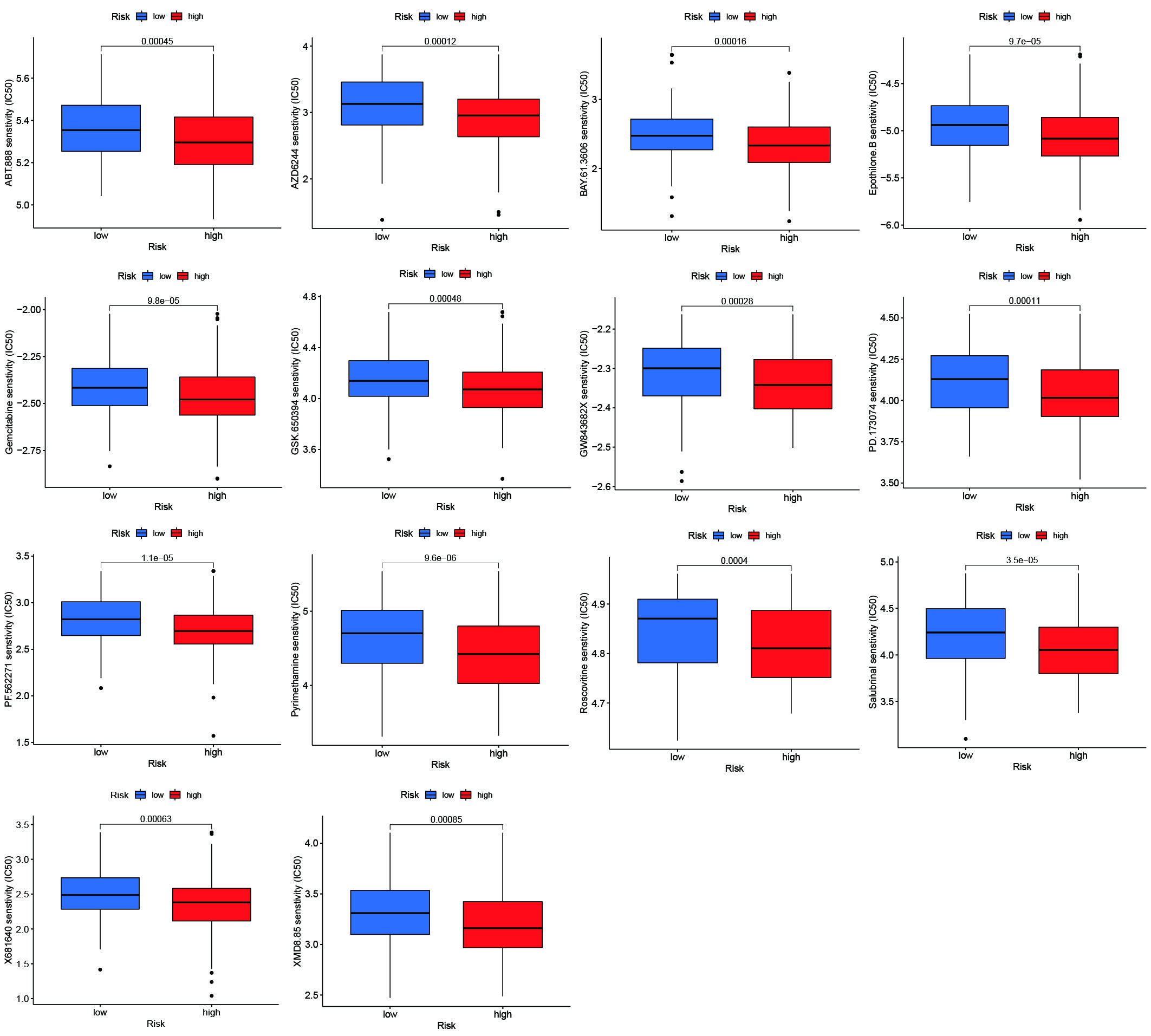

Supplement: Supplementary Figure 4 — The drug sensitivity between the low- and high-risk group (p<0.01). [file Image_4.jpeg]
