# Supplementary material for: Identification and Assessment of Necroptosis-Related Genes in Clinical Prognosis and Immune Cells in Diffuse Large B-Cell Lymphoma
Source: Front Oncol. 2022 Jun 22;12:904614. doi: 10.3389/fonc.2022.904614 (PMC9257018; doi:10.3389/fonc.2022.904614)
Supplement: Supplementary file 6 [file DataSheet_2.docx]

**Supplementary Table 3** The collinearity Tolerance and statistics VIF of each predictor.

| **Coefficients** | Collinearity Tolerance | Statistics VIF |
| --- | --- | --- |
| Risk score | 0.997 | 1.003 |
| Age | 1.000 | 1.000 |
| COO | 0.960 | 1.041 |
| LDH | 0.958 | 1.044 |

*VIF, variance inflation factor.*

**Supplementary Table 4** The collinearity diagnosis of the predictor.

| Dimension | Condition Index | Age | COO | LHD | Risk score |
| --- | --- | --- | --- | --- | --- |
| 1 | 1.000 | 0.00 | 0.02 | 0.02 | 0.02 |
| 2 | 2.733 | 0.00 | 0.03 | 0.65 | 0.25 |
| 3 | 3.064 | 0.00 | 0.54 | 0.32 | 0.25 |
| 4 | 3.713 | 0.06 | 0.39 | 0.00 | 0.46 |
| 5 | 11.395 | 0.93 | 0.01 | 0.01 | 0.02 |
